# Mechanical defensive adaptations of three Mediterranean sea urchin species

**DOI:** 10.1002/ece3.8247

**Published:** 2021-12-14

**Authors:** Konstantinos Voulgaris, Anastasios Varkoulis, Stefanos Zaoutsos, Antonios Stratakis, Dimitris Vafidis

**Affiliations:** ^1^ Department of Ichthyology and Aquatic Environment Nea Ionia, University of Thessaly Volos Greece; ^2^ Department of Energy Systems University of Thessaly Larisa Greece; ^3^ School of Mineral Resources Engineering Crete Technical University of Crete Chania Greece

**Keywords:** ecological adaptations, invasive species, mechanical properties, Mediterranean sea, mineralogy, sea urchins

## Abstract

In the Mediterranean, *Paracentrotus lividus* and *Sphaerechinus granularis* are important drivers of benthic ecosystems, often coexisting in sublittoral communities. However, the introduction of the invasive diadematoid *Diadema setosum*, which utilizes venomous spines, may affect these communities. To describe the mechanical properties of the test and spines of these three species, specimens were collected in winter of 2019 from the sublittoral zone of the Dodecanese island complex, southeastern Aegean Sea. This region serves as a gateway for invasive species to the Mediterranean Sea. Crushing test was conducted on live individuals, while 3‐point bending test was used to estimate spine stiffness. Porosity and mineralogy of the test and spine, thickness of the test, and breaking length of the spine were measured and compared, while the microstructural architecture was also determined. The test of *S*. *granularis* was the most robust (194.35 ± 59.59 N), while the spines of *D*. *setosum* (4.76 ± 2.13 GPa) exhibited highest flexibility. Increased porosity and thickness of the test were related to increased robustness, whereas increased flexibility of the spine was attributed to high porosity, indicating that porosity in the skeleton plays a key role in preventing fracture. The spines of *S*. *granularis* exhibited highest length after fracture % (71.54 ± 5.5%). *D*. *setosum* exhibited higher values of Mg concentration in the test (10%) compared with the spines (4%). For the first time, the mineralogy of an invasive species is compared with its native counterpart, while a comparison of the mechanical properties of different species of the same ecosystem also takes place. This study highlights different ways, in which sea urchins utilize their skeleton and showcases the ecological significance of these adaptations, one of which is the different ways of utilization of the skeleton for defensive purposes, while the other is the ability of *D*. *setosum* to decrease the Mg % of its skeleton degrading its mechanical properties, without compromising its defense, by depending on venomous bearing spines. This enables this species to occupy not only tropical habitats, where it is indigenous, but also temperate like the eastern Mediterranean, which it has recently invaded.

## Graphical abstract

The present work describes various defensive mechanical adaptations of a venomous invasive sea urchin species and two indigenous Mediterranean species. Furthermore, it focuses on the functional aspects of the skeleton (test, spines), revealing important differentiations among all three species, not only regarding defensive mechanisms, but also other ecological adaptations.

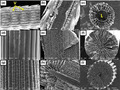

## 1 INTRODUCTION

Echinoids are a group of organisms belonging in the phylum echinodermata. They are important grazers in most marine benthic sublittoral communities (Sala et al., 1998), which can change the type of the ecosystem they live in. Predation is one of the main factors, which control their density and structure population (Shears & Babcock, 2002). To defend against predators, sea urchins utilize their calcareous skeleton, which consists mainly of the test covered by epidermis and the spines, in various ways.

Among all echinoids, diadematoids and echinothurioids are the only ones acquired with venomous spines. However, it is not yet known whether this evolutionary trait is related to decreased skeletal development, leading to decreased robustness (Koch et al., 2018). Diadematoids have also distinct skeletal morphological characteristics both for the test and for the spines (Coppard & Cambell, 2004, 2006). These may also provide them with distinct mechanical properties (Burkhardt et al., 1983).

*Diadema setosum* (Leske, 1778) diverged first from the other extant *Diadema* in the Miocene. This clade then split into two clades, one around the Arabian peninsula and the other in the Indo‐West Pacific (Lessios et al., 2001). Both environments are considered tropical, characterized by high annual temperatures. However, the recent increase in temperature of the Mediterranean Sea seems to accelerate the introduction of invasive tropical species (Bianchi, 2007). The first report about the occurrence of *D*. *setosum* in the Mediterranean dates in 2006 (Yokes & Galil, 2006). Established populations of this species have been observed in the Aegean Seas (Vafidis et al., 2021). *Paracentrotus lividus* (Lamarck, 1816) and *Sphaerechinus granularis* (Lamarck, 1816) are two species belonging to the order camarodonta, which play a key role in the sublittoral Mediterranean ecosystems. These species often coexist, with *S*. *granularis* occurring mainly in soft substrates and *P*. *lividus* on rocks and boulders (Vafidis et al., 2020). Seagrass meadows of *Zostera marina* and *Posidonia oceanica* appear to be their main habitats (Antoniadou & Vafidis, 2014).

The sea urchin skeleton is composed of ossicles made up of a trabecular meshwork, named the stereom, which in turn consists of high‐magnesium calcite (Weiner, 1985). The concentration of Mg in the calcite may differ in test and spine (Smith et al., 2016). Differences in Mg concentration seem to grant the skeletal elements different mechanical properties. Specifically, it is reported that higher magnesium concentration seems to make the structure stronger and more rigid (Weber et al., 1969). The Mg/Ca ratio is affected by temperature and salinity, meaning there might be intraspecific differences in Mg concentration in different environments (Borremans et al., 2009). Variations of the porosity of the material also seem to play a key role in its mechanical properties (elasticity, hardness, etc.; other factors like the stereom architecture may further affect these properties; Lauer et al., 2018).

The three studied species play a key role in the mid‐littoral and upper sublittoral zone. There are a limited number of studies related to the functional role of the skeleton of sea urchins, regarding the Mediterranean Sea, where attachment tenacity, spine length and thickness, and robustness of the test of *P*. *lividus*, *A*. *lixula*, or *S*. *granularis* in western Mediterranean are discussed (Collard et al., 2016; Di Giglio et al., 2020; Guidetti & Mori, 2005; Santos & Flammang, 2007). However, there are no studies regarding the eastern Mediterranean, which is characterized by peculiar oceanographic conditions. Furthermore, the study of an invasive sublittoral species, which heavily depends on its venomous spines for protection, is very important not only because it may further explain differences between mechanical and chemical adaptations but also because it is possible that this species may alter the dynamics of the Mediterranean sublittoral ecosystems, beginning from the eastern Mediterranean. This study aims to assess and explain the mechanical differences among two native camarodonts and an invasive diadematoid from an ecological standpoint, hypothesizing that *D*. *setosum* will exhibit inferior mechanical properties, as it mainly depends on its venom for protection. This might enable it to better adapt to environmental changes, as a degeneration of its skeleton due to environmental factors might not be a delimiting factor regarding its adaptive capability.

## 2 MATERIALS AND METHODS

### 2.1 Study area and collection of specimens

Living specimens of *Diadema setosum*, *Paracentrotus lividus*, and *Sphaerechinus granularis* were collected from the Dodecanese island complex, Aegean Sea, Greece, in December 2019 by scientific SCUBA diving at depths of up to 10 m. The sea bottom consisted of rocky substrates mixed with patches of sandy detritic sediments and interspersed *Posidonia oceanica* beds. An autographic conductivity temperature depth recorder, CTD (Sea Bird, Bellevue) measured temperature (20.66°C), salinity (39.37 psu), and pH (8.258). Mean test diameter and height for *D*. *setosum* (D: 66.84 ± 14.28 mm; H: 37.14 ± 8.21 mm), *P*. *lividus* (D: 59.06 ± 17.67 mm; H:51.74 ± 9.26 mm), and *S*. *granularis* (D: 76.98 ± 2.69 mm; H: 46.73 ± 4.05 mm), as well as their mean spine length (D: 72.16. ±13.79 mm; P: 16.78 ± 1.54 mm; S: 10.84 ± 0.85 mm).

### 2.2 Mechanical tests

#### 2.2.1 Test robustness

For the crushing experiment, the method of Byrne et al. (2014) was followed. Briefly, to determine the force needed to crash the test, ten live individuals from each species were crushed, using an INSTRON 3382 Universal Testing Machine of a load capability of 100 kN (22400 lbf). The device is equipped with a data acquisition system controlled from Instron Bluehill^®^ Lite Software to record load and displacement. A circular plate (15 cm diameter) was attached to the motorized tester so that an even force was applied to the test. The speed of the tester was set at 100 mm min^−1^. At the end of the experiment, the thickness of each test was measured by haphazardly selecting pieces of the test from the ambital region of each individual (*n* = 10 per species). The thickness of three pieces per individual was measured using an electronic caliper to the nearest 0.01 mm, and the average value was used in the analysis.

#### 2.2.2 3‐point bending test

To describe the stiffness of the spines, the bending modulus of elasticity (Young's modulus) was determined. The arrangement of the samples was parallel to the central axis of the spine. Ten ambital primary spines per species (one per individual) with no trace of regeneration were used for the determination of the elastic module. Force was built up with a non‐cutting blade. It was impossible to use a pin, due to the small thickness of the examined spines. The bending tests were conducted on a FTC Mechanical Testing Device 25 of a load capability of 25 KN equipped with appropriate three‐point bending grips. Feed motion was set at 5 mm s^−1^. Force (N) and deflection (mm) were monitored. The second moment of inertia (*I*, mm^4^) was determined by the software ImageJ (1.53f51), using the BoneJ plugin, taking the porous nature of the spines into account (Doube et al., 2010). The equations for the calculation of stress, strain, and Young's modulus are shown below:

$$\sigma_{f}=\frac{\mathrm{FLD}}{8I}$$

$$\varepsilon_{f}=\frac{6Dd}{L^{2}},\;E=\frac{\Delta \sigma_{f}}{\Delta \varepsilon_{f}}$$

with *σ_f_* the stress (MPa), *F* the force at fracture (*N*), *L* the active length (mm), *R* the radius of the spine at fracture (mm), *ε_f_* the strain, *D* the deflection at fracture (mm), *d* the thickness of the spine (mm), and *E* the Young's modulus (MPa). The active length for *D*. *setosum* was set at 33.75 mm, while for *P*. *lividus* and *S*. *granularis* at 10.085 mm, due to their smaller length. The length before and after fracture was measured to determine the percentage of fracture.

### 2.3 Scanning electron microscopy—Porosity

Scanning electron microscopy (SEM) was carried out on a JEOL JSM 6510. After the mechanical tests, fragments from the fracture site of the interambulacral region of the test were retrieved and together with the lower section of the fractured spines were prepared for SEM. At first, the samples were bleached to remove the soft tissues, following the method of Collard et al. (2016), NaOCl 2.5% for 1.5 h, then NaOCl 5.25% for a further 2.5 h, and air‐dried for 24–48 h. The specimens were then mounted on metal stabs with carbon‐based tape and coated with carbon. The inner surface of 10 plates and the cross and longitudinal sections of each spine (*n* = 10) were examined, using a 10 kV acceleration voltage with a 26–31 mm working distance, and secondary electron images were taken. SEM micrographs were analyzed for porosity (%) by calculating the ratio of pore area to total area using ImageJ software, as proposed by Schneider et al. (2012). The mean porosity for each species was then determined and compared. Morphological descriptions for the stereom of the tests and spines were carried out using the terminology of Smith (1980).

### 2.4 X‐ray powder diffraction analysis

An X‐ray powder diffraction analysis (XRD) of the test and spines of *D*. *setosum* was performed on a D8 Advance—BrukerAXS diffractometer using CuKα radiation to determine the crystalline phase both quantitatively and qualitatively. Interambulacral plates and spines from all individuals of *D*. *setosum* were triturated in an agate mortar until a fine state (<40 µm) and consequently pooled into one homogenous powder. Afterward, 1 g of powder was placed in a standard sample holder and was mounted in the X‐ray diffractometer. Measurements were carried out by a LynxEye detector with Ni‐filter, operated at the voltage of 35 kV, and the intensity of 35 mA, at a 2*θ* scanning range of 4–70; analyses were made at a step of 0.02^◦^ and a speed of 0.2 s per step. The evaluation of data was carried out with the Diffracplus EVA–BrukerAXS software. Identification of the experimental data was performed by fitting the diffraction pattern using Crystallography Open Database.

### 2.5 Statistical analysis

Analysis of variance (one‐way ANOVA) was used to examine differences in morphometric characteristics (i.e., test diameter, test height, test thickness, spine % length after fracture), mechanical properties (i.e., crushing force, Young's modulus), and porosity %. Prior to analysis, data were tested for normality with the Anderson–Darling test, while the homogeneity of variances was tested with Cohran's test and, when necessary, data were log‐transformed. The Tukey test was used for post hoc comparisons. ANOVAs were performed using the SPSS software package (IBM SPSS statistics v.25).

## 3 RESULTS

### 3.1 Morphological characterizations

The three species exhibit differences in their morphological features both in the test and in the spine. Not only the stereom but also their microstructures vary, but there are also similarities worth mentioning.

#### 3.1.1 Test

**FIGURE 1:**
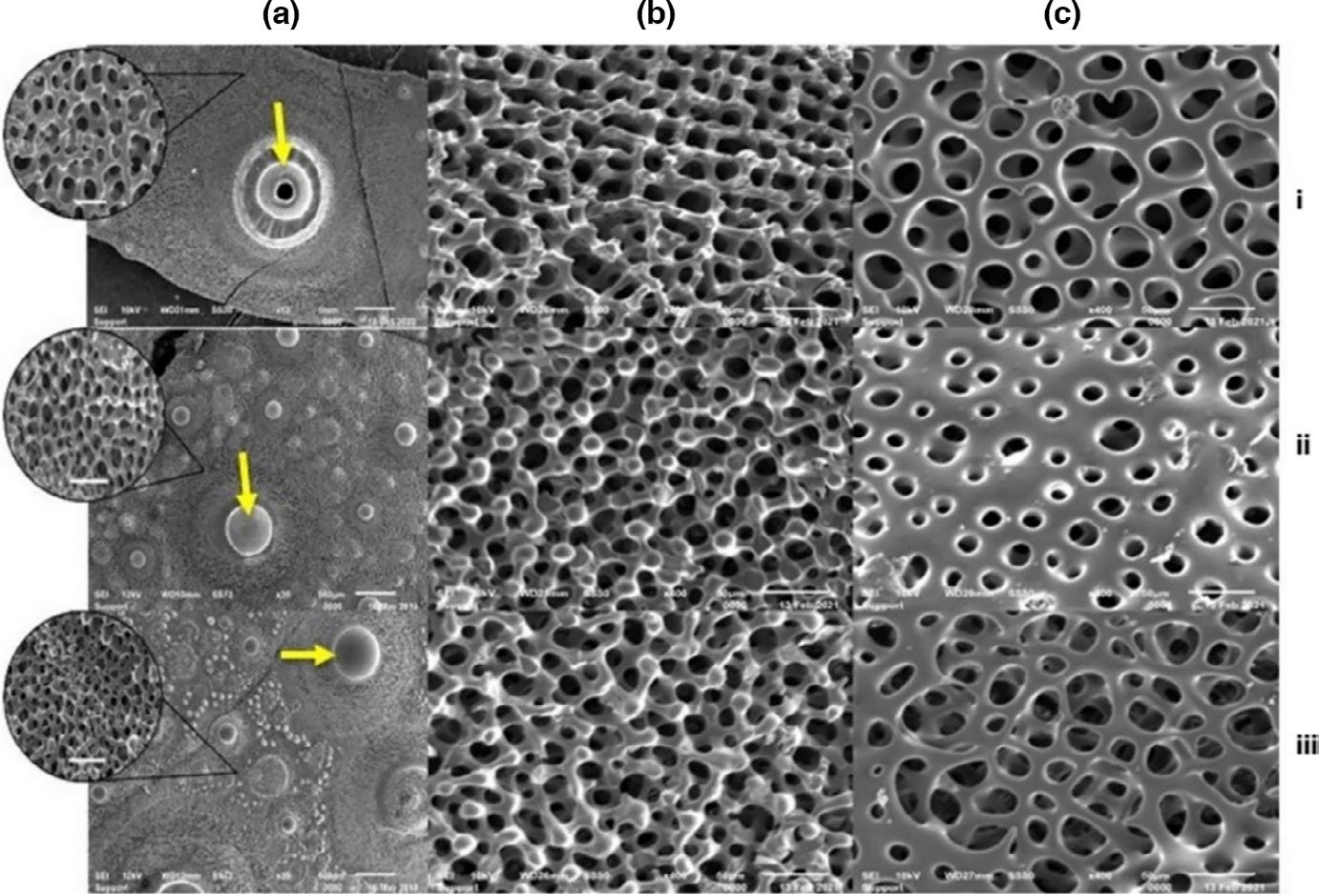
SEM micrographs of ambital interambulacral plates. (a). External surface of (i) *Diadema setosum* (scale bar‐mm, ×15), (ii) *Paracentrotus lividus*, and (iii) *Sphaerechinus granularis* (scale bar‐500 μm, ×30). Notice that the plate of *D. setosum* possesses a bigger primary tubercle and less secondary ones. Images in circles present the porous stereom of each species (scale bar‐50 μm, ×500). (b). Cross section of the same plates. (i) *D. setosum*, (ii) *P. lividus*, and *S. granularis* (scale bar‐50 μm, ×400). (c). Inner surface of the plates. (i) *D. setosum*, (ii) *P. lividus*, and (iii) *S. granularis* (scale bar‐50 μm, ×400). Vectors indicate the mamelon in each species

Observing the external surface of the interambulacral plates of the three species, morphological differences of their primary tubercles are evident (Figure 1a). *D*. *setosum* possesses perforate and crenulate tubercles, while those of the two native species appear to be imperforate and non‐crenulate. Regarding the stereom microstructure, the superficial layer seems to be different between *P*. *lividus* (dense labyrinthic) and *S*. *granularis* and *D*. *setosum* (galleried). Notice that the galleried stereom of *S*. *granularis* is denser compared to *D*. *setosum*. The middle layer of all species seems to be made of galleried stereom. The cross sections highlight the alignment of the galleries with the stereom, which follow a parallel orientation to the test surface (Figure 1b). Finally, the inner surface of the test appears denser in *P*. *lividus*, exhibiting a rather compact, simple perforate stereom. *D*. *setosum* and *S*. *granularis*, on the other hand, possess a labyrinthic inner stereom, which is rather coarse in both species but thicker in *S*. *granularis* (Figure 1c).

#### 3.1.2 Spine

**FIGURE 2:**
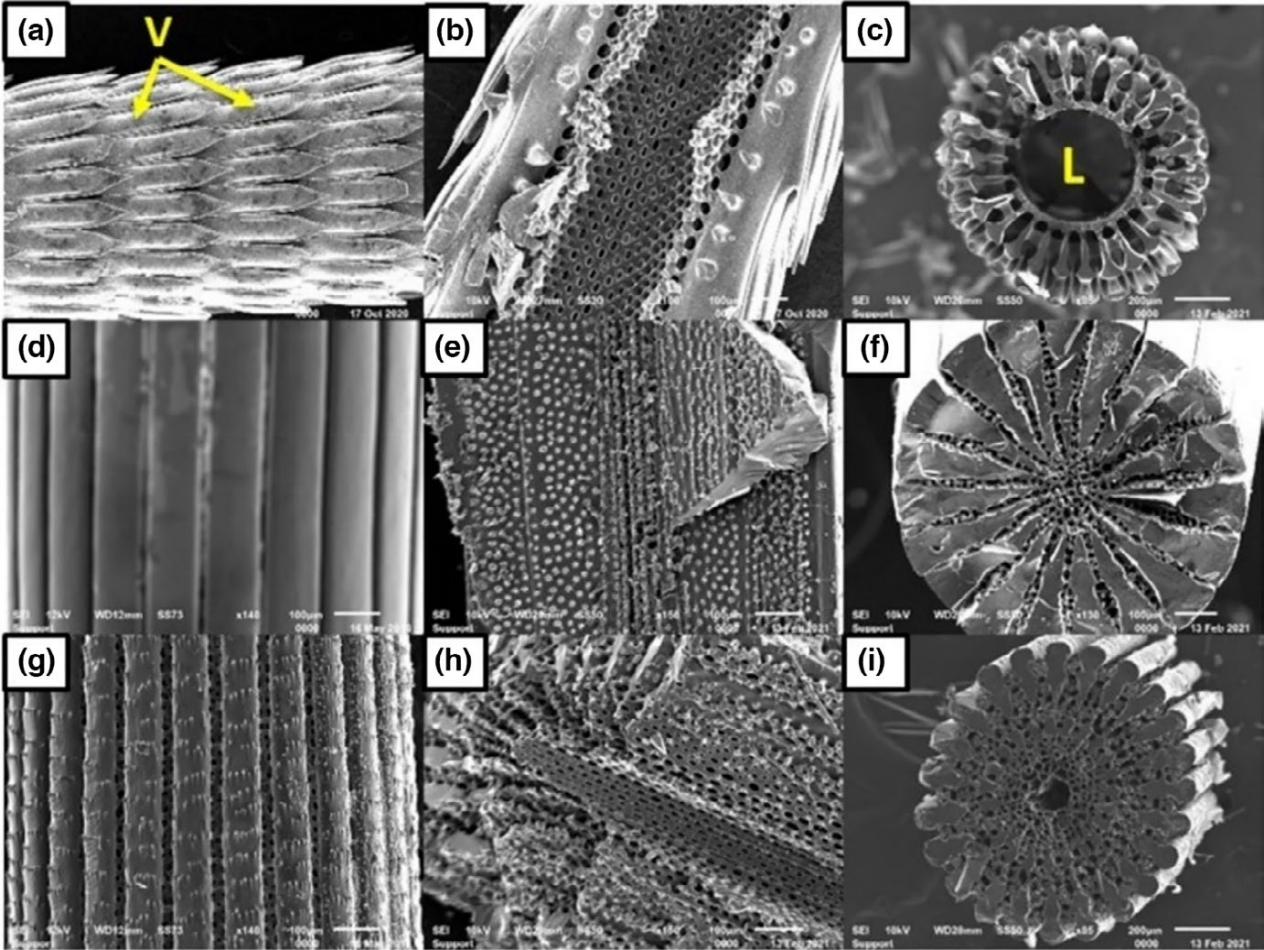
SEM micrographs of the spines of the three species. (a, d, g) External view of *Diadema setosum* (scale bar—200 μm, ×65), *Paracentrotus lividus*, and *Sphaerechinus granularis* (scale bar—100 μm, ×140). (b, e, h) Longitudinal section of *D. setosum* (scale bar—100 μm, ×100), *P. lividus* (scale bar—100 μm, ×150), and *S. granularis* (scale bar—100 μm, ×140). (c, f, i) Cross section of *D. setosum* (scale bar—200 μm, ×85), *P. lividus* (scale bar—100 μm, ×130), and *S. granularis* (scale bar—200 μm, ×85). V, verticillation; L, lumen

Concerning the texture of the external surface, differences in the spines of the three species are easily distinguishable. The verticillations of the spine of *D*. *setosum* may play a functional role since the spines of this species are difficult to remove once they pierce through skin (authors’ observation). This may enhance the chemical defense of this species through elongated time of exposure to toxin. *S*. *granularis* exhibited small, underdeveloped barbs, while the spines of *P*. *lividus* were smooth. Regarding the morphology of the spines, two main differences can be detected. First, the external surface of *P*. *lividus* is smooth (Figure 2d), while the spines of the other two species possess external accessory appendages (Figure 2a, g). However, in the case of *D*. *setosum*, these microstructures are much bigger (verticillations) and are typical of the genus *Diadema* (Figure 2a). The second main difference can be observed in cross sections of the spines (Figure 2c, f, i). The spine of *D*. *setosum* is hollow (lumen), while *P*. *lividus* is rather compact. The spine of *S*. *granularis* is acquired with an outer cortex followed by a radiating layer, which results in an open medulla. Longitudinal sections of the spines revealed a thin retiform layer of stereom in *D*. *setosum* (Figure 2b), a very confined laminar medulla in *P*. *lividus* (Figure 2e) and a laminar medulla in *S*. *granularis* surrounded by an extended galleried radiating layer (Figure 2h). It should be noted that there are thin connections between the verticillations and the thin, porous layer in *D*. *setosum* (Figure 2b) and that the wedges of *P*. *lividus* are located very close to each other (Figure 2d). Finally, the wedges of *S*. *granularis* appear to be thinner compared to those of *P*. *lividus* (Figure 2e, h).

### 3.2 Mechanical tests—Morphometrics

**FIGURE 3:**
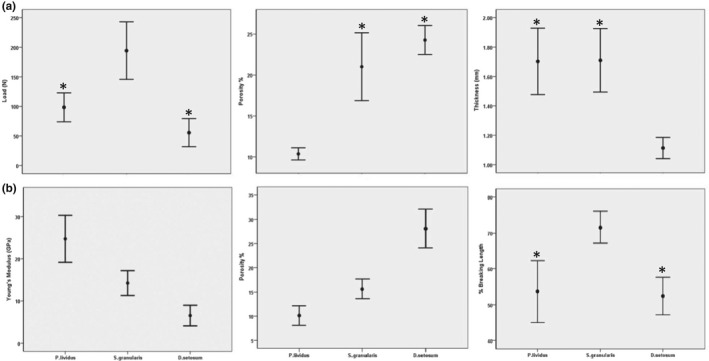
Error plots of (a): load (N), thickness (mm), and porosity (%) for the tests and (b) of Young's modulus (GPa), breaking length (%), and porosity (%) for the spines of the three species. All data are means, ±2 SE; *n* = 10. * Indicates the groups with no statistical differences

The three species exhibited significant differences for all parameters in both the test and the spines (Appendix S1). Specifically, highest force to fracture was recorded for the test of *S*. *granularis* (194.4 ± 59.6 N; *p* < .001, *F* = 16.063), while the loads of *P*. *lividus* and *D*. *setosum* did not significantly differ (98.4 ± 32.3 N and 55.6 ± 29.1 N, respectively). Regarding the porosity of the test, *P*. *lividus* exhibited significantly lower porosity (10.37 ± 0.83%; *p* < .001, *F* = 61.125), compared to *S*. *granularis* (21.02 ± 4.63%) and *D*. *setosum* (24.27 ± 1.97%). Finally, lowest values were observed for the thickness of the test of *D*. *setosum* (1.11 ± 0.08 mm; *p* = .001, *F* = 23.104), while *P*. *lividus* and *S*. *granularis* did not significantly differ (1.7 ± 0.27 mm and 1.71 ± 026 mm, respectively) (Figure 3).

**FIGURE 4:**
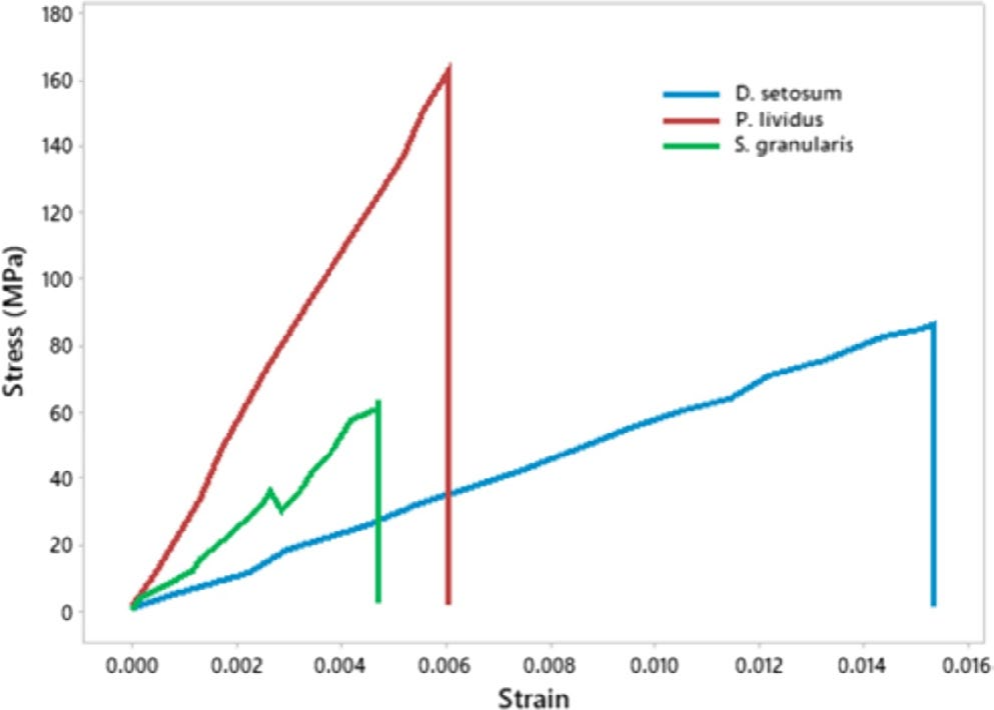
Stress–strain diagram of one representative spine per species after a three‐point bending test. The spines of *Diadema setosum* and most of the spines of *Paracentrotus lividus* exhibited linear stress increase and fracture after maximum input. On the contrary, most of the spines of *Sphaerechinus granularis* exhibited slopes indicating microfractures before the main fracture

The spines of *P*. *lividus* exhibited the highest values for Young's modulus (24.7 ± 6.2 GPa; *p* < 0.001, *F* = 21.72) and the lowest values for porosity (10.13 ± 2.03%; *p* < .001; *F* = 105.65), while *D*. *setosum* followed the reverse pattern (6.5 ± 2.7 GPa and 28.07 ± 3.98%, respectively). *S*. *granularis* showed intermediate values for both parameters, specifically 14.2 ± 3.6 GPa and 15.62 ± 2%. Finally, highest % length after fracture was observed in *S*. *granularis* (71.54 ± 5.5%; *p* = .01, *F* = 28.557), while *D*. *setosum* and *P*. *lividus* did not show any significant difference (52.5 ± 5.86% and 53.72 ± 7.41%, respectively) (Figure 3). The spines of *P*. *lividus* and *D*. *setosum* exhibited a linear force increase and breakage at maximum input. However, *S*. *granularis* showed microfractures before maximum input of load (Figure 4).

### 3.3 Mineralogy

**FIGURE 5:**
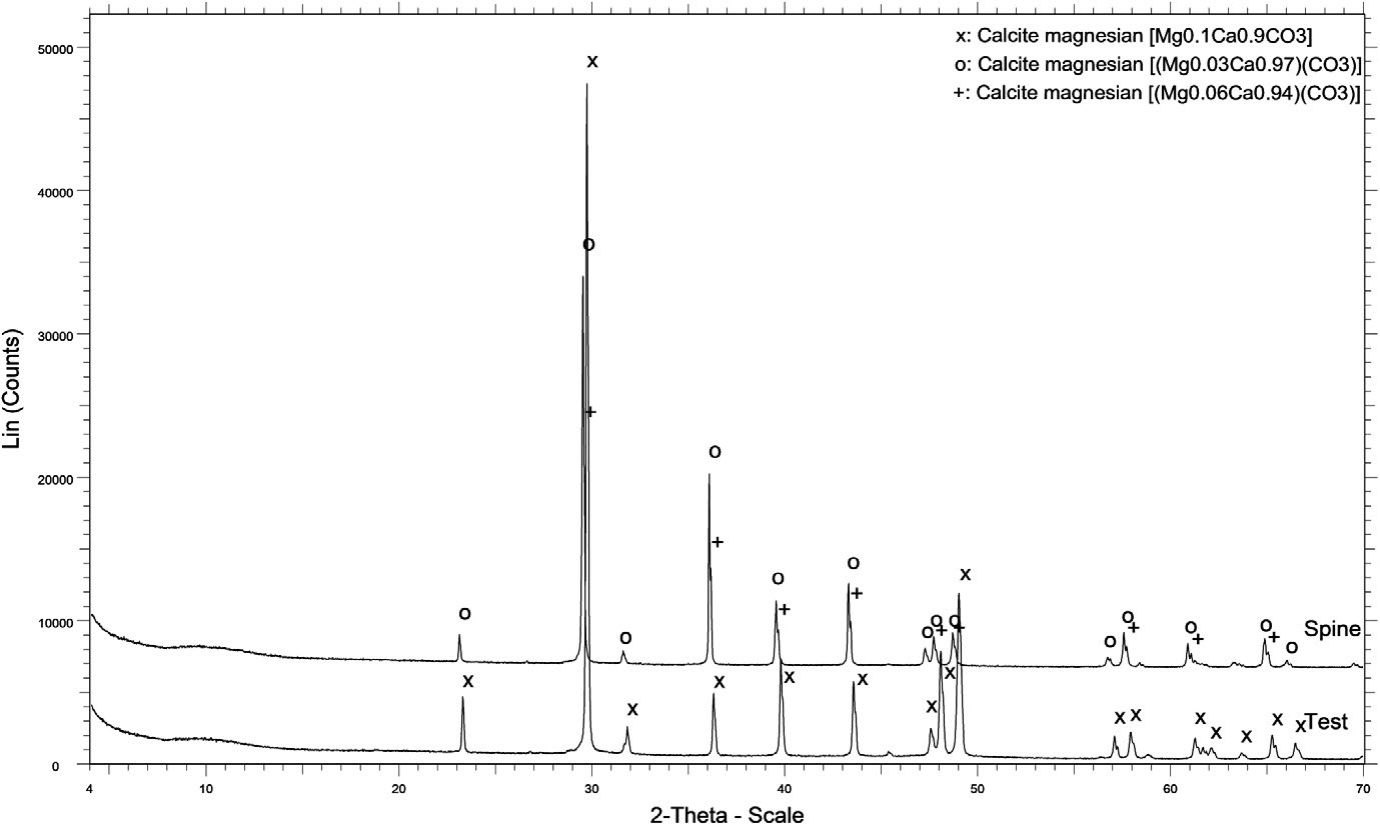
XRD spectra of the test and spine of *Diadema setosum*. Notice the three different types of peaks corresponding to different types of Mg‐ calcite, according to the Mg concentration in the crystal

X‐ray powder diffractometry showed that both the test and spines of *D*. *setosum* consist of magnesian calcite of different Mg concentrations (Figure 5). The former is made up of a single type of Mg‐ calcite containing 10% Mg, while the latter of two types. The first type contains 3% Mg participating in 70% of the total Mg‐calcite concentration, while the second type is made up of 6% Mg, resulting in a mean concentration of 4% Mg in the spines of *D*. *setosum*. The Mg percentage of the calcite in the test reaches 10%, while that of the spine was detected in two phases, specifically one that contains 3% Mg and another with 6% Mg. Quantitatively, the spine is made up of 70% of the first phase (3% Mg) and 30% of the second phase (6% Mg). Thus, the mineral composition of the spine of *D*. *setosum* is characterized by heterogeneity.

## 4 DISCUSSION

### 4.1 Test and spine morphology

Comparison of SEM micrographs showed differences in the micromorphology of test and spines among the three species. It should be noted that many previous studies have been concerned with the skeletal micromorphology of the examined species (Burkhardt et al., 1983; Régis, 1979, 1986; Regis & Thomassin, 1982; Smith, 1984), although the examined specimens did not originate from the eastern Mediterranean Sea. However, no interspecific differences were observed regarding the type of the trabecular meshwork. External differences regarding the test include the tubercles, which, in *D*. *setosum* are perforate and crenulate, a characteristic feature of the genus *Diadema*. This perforation serves as a pathway for the central ligament to penetrate the spine and may provide increased mobility of the spine as pointed out by Motokawa (1983). Moreover, since the spines of this species are long in relation to its test, crenulation of the tubercles serves as attachment of a thick and more developed catch connective tissue (Motokawa & Fuchigami, 2015) which also accounts for the rapid movements of the spines. The two native species, one the other hand, have solid non‐crenulated tubercles. These may provide the ball socket joint with more stability. In other words, *D*. *setosum* presents perforation leading to increased mobility and crenulation to possibly increase the angular range of motion of the spine. On the other hand, the two camarodonts, lacking venomous spines, do not exhibit perforations. Instead, during impact the ball socket joint needs to remail firm, so smooth tubercles may be most suitable. Since the catch connective tissue plays a key role in changing the mechanical properties of the ball socket joint (Wilkie, 1996), the functional morphology of the tubercles is directly related to the activity and mutability of this tissue. More research on this aspect needs to be conducted, as it is evident that different species use spines for different purposes and in different ways.

Regarding the stereom of the test of the three species, that of *P*. *lividus* appears to be denser both externally and internally, with a compact superficial labyrinthic stereom, a dense galleried middle layer and a perforate inner surface. *D*. *setosum* and *S*. *granularis* exhibited galleried stereom superficially and in the middle layer and a labyrinthic inner surface. The stereom of *D*. *setosum*, however, seems to be generally more open than that of *S*. *granularis*.

Furthermore, morphological differences among the spines of the three examined species seem to be related to different adaptations. *D*. *setosum* utilizes its spines so that the efficiency of its toxins is maximized. Increased elasticity, which is attributed to a highly porous stereom, a lumen through which venom is secreted and verticillations on the outer surface play a key role in achieving successful defense against predation. On the other hand, *P*. *lividus* aims to inflict maximum mechanical damage to possible predators using stiff, sharp‐edged spines. Stiffness is achieved by thick septa and decreased porosity, while the spines of this species are devoid of any external micro‐appendages (barbs, verticillations, etc.), possibly enabling the spine to pierce threw flesh as deep as possible. Finally, *S*. *granularis* does not possess pointy spines, which indicates that its defense does not involve piercing the predator. Rather than that this species utilizes its spines to minimize the impact generated by an attack on the test, and this seems to be the main reason why this species prefers maintaining most of the spine during fracture, rather than breaking it. Thus, the spines of S. granularis function in such a way that the impact is spread among as many spines as possible, resulting in reduced pressure per spine. For that purpose, long, brittle spines would not be suitable. External barbs seem to play a role in maximizing the distribution of the impact inflicted on the spine, by providing “crack deflecting horizons” and thus increasing energy absorption during impact, as proposed by Presser et al. (2009) for the dome‐shaped marks of the spine of Heterocentrotus mammillatus. Finally, porosity also seems to complement the function of the spines by providing them with flexibility.

### 4.2 Robustness of test—Stiffness of the spine

The test of *S*. *granularis* appears to be able to withstand significantly more load than the other two species. Guidetti and Mori (2005) found the test robustness of *P*. *lividus* to range from 11.57 (1180 g) to 109.64 N (11180 g), while test thickness ranged from 0.12 to 0.90 mm. Present results exhibit higher values for *P*. *lividus* both for the load and thickness, meaning that an increase in thickness results in a more robust test in the case of *P*. *lividus*. *D*. *setosum* and *P*. *lividus* did not exhibit any significant differences in their ability to withstand load, but the test of the former was thinner and more porous. This indicates that this species builds less material but in a more efficient way and that porosity may play a more important role than thickness in the mechanical design of the test of this species. It is important to note that the mechanical properties of the skeleton of the test are not the only factor that determines the strength of the organism against a potential mechanical attack. Ellers et al. (1998) showed that the sutures that hold the skeleton in place play a key role in the mechanical tenacity of the test. These sutures are comprised of soft tissue, specifically collagenous mutable connective tissue, and provide the test with more strength. *D*. *setosum* may utilize these sutures to gain robustness, making up for its decreased thickness. However, this study is meant to deal exclusively with the mechanical properties of the skeleton and how these are utilized by the three examined species for different purposes.

The Young's modulus of the spines was significantly different for all species with *P*. *lividus* being the stiffest and *D*. *setosum* the most elastic. Burkhardt et al. (1983) carried out a cantilever‐beam experiment on the spines of *D*. *setosum* and *D*. *antilarum* both fresh and treated and found the Young's modulus of 52.1 ± 9.5 GPa for fresh spines. The origin of the individuals used is not reported. The differences in reported values may lay in the different methods followed. Moureaux et al. (2011) reported values of 22 GPa for the spines of *P*. *lividus*, using the cantilever‐beam method as well. The same study reported values of 58.57 ± 3.76 for the septa and 32.20 ± 3.26 GPa for the central stereom of the spines through nanoindentation. In the present study, the porosity seems to follow an inverse trend to Young's modulus, indicating that porosity in the spine accounts for greater elasticity. The spines of *D*. *setosum* and *P*. *lividus* appear to break near the middle of the spine with 52.5% and 53.72% of the spine remaining intact after fracture. *S*. *granularis* exhibited higher values, around 71.54% of the total length. This may show a tendency of *S*. *granularis* to maintain its spines rather than break them, possibly following a different adaptation against predation.

It must be noted that being a toxopneustid, *S*. *granularis* possesses globiferous pedicellaria, which are venomous defensive appendages. Thus, this species exhibits an intermediate ecological adaptation, depending both on its skeleton and chemical defense provided by the pedicellaria for its defense, as proposed by Ghyoot et al. (1994). Finally, *D*. *setosum* and *P*. *lividus* seem to follow a linear increase in stress until fracture, but *S*. *granularis* exhibited slopes indicating the occurrence of microfractures possibly related to the presence of external barbs. This characteristic may further enhance the tendency of this species to prevent its spines from breaking (Figure 4). This species may utilize its spines complementarily to the increased robustness of the test. Thus, we theorize that *S*. *granularis* develops its skeleton in such a way, as to be able to withstand high amounts of impact and possibly inhabit soft substrates more effectively, maintaining its spines rather than breaking them as a kinetic adaptation on sandy substrates. Another explanation might be that *S*. *granularis* prefers to inhabit soft substrates, to further minimize pressure on the test, by utilizing sandy substrates for the absorption impact. To accomplish this feat, it is necessary for its spines to maintain most of their length after fracture. Finally, it must be noted that 3‐point bending might not mimic the way spines break during predation. However, it is a suitable method for comparing biogenic hard material (shells, bones, etc.) with technical samples (Reilly & Currey, 1999).

### 4.3 Mineralogy

The test of *D*. *setosum* contains higher Mg concentration in the calcite compared to the spines. It is also noteworthy that there are two types of Mg‐calcite in the spines of *D*. *setosum*. This might indicate an intraspecific diversity of the calcite concentration of the spines, either among different individuals or among different areas of the spine (e.g., base, shaft, tip). This species exhibits intermediate values of Mg concentrations in the calcite both for the test and spines (Table 1) compared to the two native ones. Ma et al. (2009) concluded that the increase of Mg concentration increases the strength of the teeth of *P*. *lividus*. However, Mg concentration in the calcite does not seem to contribute to the interspecific mechanical differences regarding both in the test and in the spine. Differences among species regarding the homogeneity of Mg content within a single skeletal element, however, may exist as described in Ma et al. (2009), where Mg increased toward the tip of the tooth.

**TABLE 1:** Mg content % in calcite of the test and spine regarding the three studied species

Species	Area	MgCO3 (% in calcite)		References
		Test	Spine	
*P. lividus*	Pagasitikos (Mediterranean)	12.9	3	Varkoulis et al. (2020)
*P. lividus*	Rethimnon (Mediterranean)	–	3.9	Magdans and Gies (2004)
*P. lividus*	Gulf of Lion (Mediterranean)	11.02	3.6	Mcclintock et al. (2017)
*P. lividus*	Lesvos (Mediterranean)	–	2.9	Richter (1984)
*P. lividus*	Caesarea (Mediterranean)	9.7	2	Raz et al. (2000)
*S. granularis*	Pagasitikos (Mediterranean)	6	6	Varkoulis et al. (2020)
*D. setosum*	Dodecanese (Mediterranean)	10	4	Present study
*D. setosum*	Fiji (Pacific)	14.8	–	Ebert (2001)
*D. setosum*	Guam (Pacific)	13.5	12.2	Weber et al. (1969)

The results seem to confirm the hypothesis that increasing temperature leads to increased Mg concentration, since *D*. *setosum* in temperate waters (Mediterranean) exhibited lower values of Mg compared with those in tropical waters (Pacific) both for the test and spines (Smith et al., 2016) (Table 1). This difference is important since the values reported in the present study are lower than the threshold above which high Mg‐calcite becomes more soluble than aragonite (Bischoff et al., 1987). Numerous studies have been conducted describing the Mg % in the calcite regarding *P*. *lividus*, ranging between 11.02 and 12.9% for the test and 2.9 to 3.9% for the spine (Table 1). However, there is only one study regarding the Mg concentration in the skeleton of *S*. *granularis*. This species exhibits the highest Mg concentration among the three studied echinoids regarding the spine and lowest regarding the test (Table 1). The fact that the Mg concentration is not interspecifically correlated with robustness in the test or stiffness in the spine may indicate different utilization patterns of Mg in the skeleton of echinoids. This is the first study comparing an invasive species in the Mediterranean with its counterpart in the tropic zone, showing the effect of environmental factors on the Mg concentration in the calcite. The energetic aspects of Mg maintenance in the sea urchin skeleton are not yet adequately understood. It may be possible that the chemical defense of *D*. *setosum* may enable this species to inhabit different environments, as it is not solely dependent on the mechanical properties of its skeleton. The dominant principle regarding toxic secretions is that they serve as ecological adaptations in order for the organism to gain a competitive advantage either against predators or competitors (Casewell et al., 2013). *D*. *setosum* has invaded the Mediterranean, where temperatures are lower compared with its original habitats in the tropics. Even though decreased temperature and pH may lead to decreased robustness of the skeleton, these factors are not limiting the distribution of *D*. *setosum*, arguably because this species depends more on its toxic secretions than on its mechanical defense.

*Diadema setosum* uses venomous, brittle spines as its main defensive strategy. The thinner, less compact test, as well as the hollow spine indicate that this type of defense may be metabolically expensive. However, this species may compensate, in that its skeleton might be lighter, resulting to increased mobility. Furthermore, the hollow spines of these species may be less expensive to regenerate. The two native species, on the other hand, use different types of mechanical defense. This may be a result of *S*. *granularis* possessing globiferous pedicellaria and thus a partial type of chemical defense.

## 5 CONCLUSION

The species examined in the present study are two common camarodonts of the eastern Mediterranean and one invasive diadematoid. Micromorphological aspects, morphometrical features, mechanical properties, and skeletal mineralogy were compared in order to explain not only the defensive adaptations of these species, but also the invasion of *D*. *setosum* in the eastern Mediterranean Sea. It is evident that this species depends on its venomous baring spines in order to be able to inhabit environments of lower temperature, where the skeletal Mg concentration is decreased compared to the tropics, leading to degraded mechanical properties. In this way, it does not compromise its defense against predation. *S*. *granularis* is acquired with globiferous pedicellariae, which provide a level of chemical defense, but does also depend on its highly robust test to withstand mechanical impacts. Furthermore, its obtuse spines exhibit a tendency to maintain most of their length after fracture, which may have led to this species being able to inhabit soft substrates. *P*. *lividus* mainly depends on its mechanical properties to defend against predation. This might be linked with lower energetic costs, as the production of toxins is known to be metabolically expensive. This is the first study to deal with a direct comparison of three different sea urchin species in terms of how differences in their mechanical properties might explain their different defensive adaptations. However, there are certain aspects that need to be further examined, the most important being the energy costs of these defensive mechanisms, and how they affect the population dynamics of this species. Furthermore, more research needs to be conducted as to how soft tissue affects the mechanical properties of sea urchins in an interspecific level.

## ACKNOWLEDGMENTS

The authors thank Anastasios Koutsidis for his technical contributions during the mechanical experiments.

## CONFLICT OF INTEREST

The authors declare no conflicts of interest.

## AUTHOR CONTRIBUTIONS

**Konstantinos Voulgaris:** Conceptualization (lead); Methodology (lead); Software (lead); Visualization (lead); Writing‐original draft (lead); Writing‐review & editing (lead). **Anastasios Varkoulis:** Conceptualization (lead); Data curation (lead); Formal analysis (lead); Methodology (lead); Software (lead); Visualization (lead); Writing‐original draft (lead); Writing‐review & editing (lead). **Stefanos Zaoutsos:** Data curation (supporting); Methodology (equal). **Antonios Stratakis:** Methodology (equal). **Dimitris Vafidis:** Conceptualization (lead); Formal analysis (supporting); Project administration (lead); Supervision (lead); Visualization (equal); Writing‐review & editing (supporting).

## Supporting information

Appendix S1

## Data Availability

The data used for this manuscript are uploaded to Dryad: https://doi.org/10.5061/dryad.d51c5b03b.
