# Supplementary material for: Mechanical defensive adaptations of three Mediterranean sea urchin species
Source: Ecol Evol. 2021 Dec 14;11(24):17734–43. doi: 10.1002/ece3.8247 (PMC8717311; doi:10.1002/ece3.8247)
Supplement: Supplementary file 1 — Appendix S1 [file ECE3-11-17734-s001.docx]

**Apendix**

***Table. ANOVA results of the morphometric and mechanical parameters of the test and spines among the three species. (F—variance of the group means for ANOVA; df—Degrees of freedom; p: probability value).***

|  | | df | F | Sig.(p) | Sum of Squares | Mean Square |
| --- | --- | --- | --- | --- | --- | --- |
| **Test** |  |  |  |  |  |  |
| Load | Between Groups | 2 | 16.063 | .000 | 100991.7 | 50495.8 |
|  | Within Groups | 27 |  |  | 54335.5 | 2012.43 |
|  | Total | 29 |  |  | 155327.2 |  |
| Porosity | Between Groups | 2 | 61.125 | .000 | 1057.32 | 528.66 |
|  | Within Groups | 27 |  |  | 280.51 | 10.39 |
|  | Total | 29 |  |  | 1337.83 |  |
| Thickness | Between Groups | 2 | 23.104 | .001 | 2.36 | 1.18 |
|  | Within Groups | 27 |  |  | 8.53 | 0.32 |
|  | Total | 29 |  |  | 10.89 |  |
| **Spines** |  |  |  |  |  |  |
| Y.modulus | Between Groups | 2 | 21.72 | .000 | 1704.8 | 852.4 |
|  | Within Groups | 27 |  |  | 593.82 | 21.99 |
|  | Total | 29 |  |  | 2298.62 |  |
| Porosity | Between Groups | 2 | 105.65 | .000 | 1689.01 | 844.51 |
|  | Within Groups | 27 |  |  | 269.87 | 9.99 |
|  | Total | 29 |  |  | 1958.89 |  |
| % Length | Between Groups | 2 | 28.557 | .001 | 2274.19 | 1137.09 |
|  | Within Groups | 27 |  |  | 1074.72 | 39.80 |
|  | Total | 29 |  |  | 3348.91 |  |
